# Patient education program for Brazilians living with diabetes and prediabetes: findings from a development study

**DOI:** 10.1186/s12889-021-11300-y

**Published:** 2021-06-26

**Authors:** Gabriela Lima de Melo Ghisi, Mariana Balbi Seixas, Daniele Sirineu Pereira, Ligia Loiola Cisneros, Danielle Guedes Andrade Ezequiel, Crystal Aultman, Nicole Sandison, Paul Oh, Lilian Pinto da Silva

**Affiliations:** 1 Cardiovascular Prevention and Rehabilitation Program, Toronto Rehabilitation Institute, University Health Network, Toronto, M4G 1R7 ON Canada; 2 Faculty of Physical Therapy, Federal University of Juiz de Fora, Juiz de Fora, MG 36038-330 Brazil; 3 Department of Physiotherapy, Federal University of Minas Gerais, Belo Horizonte, 31270-901 MG Brazil; 4 Faculty of Medicine, Federal University of Juiz de Fora, Juiz de Fora, 36038-330 MG Brazil

**Keywords:** Diabetes mellitus, Needs assessment, Patient education, Prediabetes state, Self-management

## Abstract

**Background:**

Globally, the incidence of diabetes is increasing and strategies to reach a comprehensive approach of care are needed, including education in self-management. This is particularly true in low and middle-income countries where the number of people living with diabetes is higher than in the high-income ones. This article describes the development of a structured patient education program for Brazilians living with diabetes or prediabetes.

**Methods:**

These steps were undertaken: 1) a 4-phase needs assessment (literature search of local diabetes guidelines, environmental scan, evaluation of information needs of patients identified by diabetes experts, and patient focus groups); and, 2) the translation and cultural adaptation of the patient guide (preparation, translation, back-translation, back-translation review, harmonization, and proofreading).

**Results:**

Four of the seven guidelines identified include educational aspects of diabetes management. No structured education program was reported from the environmental scan. Regarding the information needs, 15 diabetes experts identified their patients’ needs, who referred that they have high information needs for topics related to their health condition. Finally, results from six patient focus groups were clustered into six themes (self-management, physical activity, eating habits, diabetes medication, psychosocial being, and sleep), all embedded into the new education program. Constructive theory, adult learning principles, and the Health Action Process Approach model were used in program development and will be used in delivery. The developed program consists of 18 educational sessions strategically mapped and sequenced to support the program learning outcomes and a patient guide with 17 chapters organized into five sections, matched with weekly lectures.

**Conclusions:**

This program is a sequential and theoretical strategic intervention that can reach programs in Brazil to support diabetes and prediabetes patient education.

**Supplementary Information:**

The online version contains supplementary material available at 10.1186/s12889-021-11300-y.

## Background

In the past years, the care of people with diabetes evolved from being purely controlling blood glucose and risk factors (blood pressure, hyperlipidemia, and smoking) to a more comprehensive approach, including education in self-management [1, 2]. The United States Standards for Diabetes Self-Management Education and Support define diabetes self-management as “a collaborative and ongoing process intended to facilitate the development of knowledge, skills, and abilities that are required for successful self-management of diabetes” [3]. Education in diabetes should not only focus on increasing patients’ knowledge about their health condition, but also promoting lifestyle changes [4–7]. Particularly, lifestyle changes related to the adherence of medications, increase of physical activity levels, adoption of a healthy diet, positive changes in psychosocial issues, better clinical outcomes (e.g., weight, glycated hemoglobin (HbA1c), lipid levels, smoking), and ultimately an improvement in the quality of life [4–6].

A structured education program for people with diabetes should include the following components: based on evidence and patients’ needs; have clear learning objectives; support not only patients but their families and caregivers in developing attitudes, beliefs, knowledge, and skills to self-manage diabetes; have supported materials; and be delivered by trained educators [3, 8]. The real goal of education in diabetes should always be to improve their self-efficacy (individual’s perceptions or beliefs about how capable he or she is of performing a specific activity or task) [9] and, accordingly, their self-management ability. This change can help patients to navigate through daily challenges in their care and ultimately promote short- and long-term quality of life [3, 5, 6].

Evidence from randomized controlled trials and observational studies suggest that education in diabetes management is cost-effective [10, 11], improves knowledge [12–14], clinical outcomes [12, 13, 15, 16] – including significant decreases in HbA1c levels [17, 18] and improvements in gamma-hydroxybutyrate (GHb) levels [4], psychosocial outcomes [13, 19, 20] and quality of life [6, 21–23]. However, the characteristics of these programs are inconsistent, and even studies have shown that mode of delivery and hours of engagement may influence outcomes [18], there is no consensus on the best approach to deliver education to these patients.

Currently, many countries address patient education as a critical element of care for all people with diabetes and those at risk of developing the disease. In high-income countries, education in self-management has become an essential part of the approach to managing type 2 diabetes. As an example, the United States government has supported a national diabetes program that aims to increase the number of diabetes patients that receive formal education by 60% [24]. Other initiatives are happening in the United Kingdom, including the recommendation from the National Institute for Health and Care Excellence (NICE) [8]. The NICE recommends that well-designed and well-implemented educational programs are cost-effective for people with diabetes and should be offered to every person around the time of diagnosis with annual reinforcement and reviews [8].

Despite barriers to healthcare access, low-income countries are also developing structured programs for diabetes care that include education. An example is Bangladesh, where the Diabetic Association of Bangladesh (DAB) has developed a decentralized model with 61 affiliated associations that are looking after 35% of all people with diabetes in the country [25]. This association has developed a month-long certificate course for diabetes educators, and approximately 150 educators have completed it. Findings from studies in low- and middle-income countries like the Philippines [26], Iran [27], and South Africa [28] support the effectiveness of implementing a diabetes education program to improve the care of these patients. To our knowledge, there are no similar efforts in South American countries, including Brazil. Therefore, the objective of this article is to describe the development of a patient education program for Brazilians with diabetes and prediabetes. We hypothesize that following the steps presented here we will be able to develop a culturally adapted and effective intervention for the assigned population.

## Methods

### Context

Diabetes College™ [29] is a standardized, comprehensive, evidence-based, online, multi-media patient education intervention freely-available worldwide. It was developed through a rigorous process, informed by a theorethical foundation [30–34], in conjunction with patient partners and global diabetes experts. The program specifically aims to help people treat diabetes, get active, eat healthy, feel well, and take control of their health, and these are the five pillars (i.e. content areas) of the program.

Diabetes College was originally created in English (2016) as part of the Diabetes Program at Toronto Rehabilitation Institute in Toronto, Canada. Diabetes College is delivered mainly through two main tools - a website and a patient guide. The patient guide (entitled “*A Guide to Help You Live and Thrive with Diabetes*”) is written in plain language, and has 17 chapters. The effectiveness of this education intervention has been confirmed by a previous study, with significant improvements in patients’ knowledge, exercise, food intake, self-efficacy, and health literacy [35].

### Phases of patient education program development for people living with diabetes or prediabetes in Brazil

The development of the patient education program involved a rigorous 2-year process with multiple steps of research, analysis, and revision. It envolved quantitative and quantitative analysis, in order to achieve our goal of developing a culturally adapted and effective intervention for diabetes and prediabetes patients in Brazil. This study adheres to STROBE guidelines where applicable.

#### Needs analysis

The first phase was a needs analysis, which has been highlighted in the literature as one of the essential stages in the process of developing an education program [36]. The assessment of needs is multifactorial and involves patients, healthcare providers, and the environment. The needs analysis as part of this program development process included the four steps described below.

- Literature review of best practice on diabetes patient education: a literature search of current (last 10 years) diabetes guidelines from Brazil and South America was conducted for information regarding patient education. Guidelines from Brazilian and South American Societies of Diabetes and Rehabilitation were included in the search, as well as a snowball search was performed using the terms “diabetes guidelines”, “South America”, “Brazil”, “diabetes education”, “health education”, “education guidelines”. Data were extracted and combined into categories following the standards of reporting of behavior change interventions provided by the Workgroup for Intervention Development and Evaluation Research (WIDER) [37]: characteristics of those delivering the intervention (i.e., type of healthcare professional), recipients characteristics, the setting (i.e., time and place of intervention), delivery mode, intensity (i.e., number of sessions), duration (i.e., each session duration), adherence to delivery protocols, and a detailed description of the intervention content.
- Environmental scan of other educational programs for diabetes patients: to understand how education is delivered to people with diabetes, an environmental scan was performed. Cardiac rehabilitation (CR) centers identified by a previous study [38] in two Brazilian cities were included in this scan. These centers were contacted by email, and program managers were asked to answer eight questions related to their educational programs’ characteristics based on the WIDER [37]. If no response was received within a week, the same message was sent once again (see Additional file 1).
- Assessment of information needs of patients identified by diabetes experts: based on a validated tool to assess the information needs of cardiac patients [39] and their healthcare providers [40], an instrument was developed to evaluate the information needs of patients to be completed by a convenience sample of diabetes experts. The instrument comprised 80 items – based on Diabetes College content – where experts were asked to rate the importance of each topic for diabetes patients from 1 = *really not important* to 5 = *very important*. This instrument also included one multiple-choice question related to how they identified that the patients prefer the education topics to be delivered (see Additional file 2).
- Patient focus group: focus groups are collective discussions that are designed to explore a specific set of issues. They are a qualitative technique appropriate for exploring patients’ knowledge and experiences, examining not only what they think but why they hold a particular opinion. They can reveal dimensions of understanding that often remain untapped by quantitative data methods. Thus, focus groups explore people’s perception of issues by encouraging interaction between research participants [41]. Six focus groups were undertaken in two Brazilian cities to determine the patients’ experience living with diabetes or prediabetes, and procedures followed in conducting it are detailed in an additional file (see Additional file 3). Focus group sessions were digitally audio-recorded and transcribed verbatim. An iterative and reflexive thematic content analysis was used to identify, analyze and report themes evolving from the focus groups [41, 42].
- Patients’ narratives were coded using a qualitative framework and grouped under themes [41]. Sample size was based on the principle of theoretical saturation, a process by which data are collected and analyzed until data saturation is reached. This is defined as the point at which no new themes emerged.

#### Translation and cultural adaptation of the diabetes college patient guide

The final phase of the development of this patient education program was the translation of the patient guide from the original language (English) to Brazilian Portuguese and the adaptation of the content based on the analysis of the information about needs obtained in the steps previously described. The patient guide is the primary tool for this program and was the focus of the translation and cultural adaptation process. This phase followed guidelines for best practice to ensure the information was accurate, culturally sensitive, and the translation of high quality [43–47]. The process was divided into six steps: (1) preparation, (2) translation, (3) back-translation, (4) back-translation review, (5) harmonization, and (6) proofreading. Cultural adaptation of the materials was embedded in this process and performed following best practices on cultural adaptation [46–48]. Written informed consent was obtained from all participants.

## Results

### Literature review of best practice on diabetes patient education

In regards to the needs analysis, the first step was a literature review of best practices on diabetes patient education in Brazil and South America. Seven guidelines were found, of which four included educational aspects of diabetes management [1, 49–51]. None of the documents reported all eight characteristics of the educational intervention following the standards of reporting of behavior change intervention provided by the Workgroup for Intervention Development and Evaluation Research (WIDER), which providers recommendations for reporting components of behavior change interventions [37]. Table 1 summarizes the results from this literature review.

**Table 1:** Summary of literature review of best practice in diabetes patient education (*n* = 4)

Guidelines (author, year)	Characteristics of those delivering the intervention	Characteristics of the recipients	The setting	Mode of delivery	The intensity	The duration	Adherence to delivery protocols	Intervention content
Brazilian Ministry of Health, 2013 [52]	Any healthcare professional.	Not provided.	Not provided.	Not provided.	Not provided.	Do not specify the number of sessions but mention that it should be continuous and start at the first consultation.	Not provided.	Specific to foot care (ulcers).
Brazilian Society of Diabetes, 2014 [1]	Any healthcare professional, but it should include a qualified diabetes educator.	Not provided.	Not provided.	Physical space and features include the following: - privacy and confidentiality - comfortable seats, lighting, and air quality - safe environment (free from any dangers) - waiting rooms and toilets should be provided - accessibility for people with physical disabilities - teaching tools, communication technology and the proper equipment to support the multi-professional team must be available and include the following: - adequate audiovisual resources - telephone and fax services - office supplies and equipment - record-keeping system - computer and internet access	Not provided.	Do not specify the number of sessions but mention that it should be continuous for better results.	Not provided.	- Eat healthily - Practice regular physical activity - Check insulin levels - Take medications - Solve problems - Reduce risk factors - Adapt life in a healthy way.
Brazilian Society of Diabetes, 2019–2020 [53]	Healthcare providers and teams qualified in diabetes education: doctors, physical educators, dietitians, nurses, psychologists, social workers, pharmacists, and dentists.	Children and adolescents living with type 1 diabetes	Not provided.	Activities in groups, workshops, and lectures. Nutritional orientation groups. Dialogical, reflexive, and critical perspectives can be an effective instrument for the formation of critical knowledge. Group dynamics, personal experiences, games, forums, and webpages.	Not provided.	Not provided.	Not provided.	The five behavior change stages of the Transtheoretical Model proposed by Prochaska: pre-contemplation, contemplation, preparation, action, and maintenance. Eating plan, adequate physical exercise, self-care practices to reduce risk factors, motivating techniques to live with diabetes. The Agency for Healthcare Research and Quality (AHRQ) recommends that the following areas of knowledge should be reviewed and/or advised before patient discharge (if applicable): - identify the team that will continue the post-discharge patient follow-up - diagnosis, self-monitoring and glycemic goals - definition, recognition, treatment, and prevention of hyperglycemia and hypoglycemia - nutritional habits - diabetes medicines: when and how (oral and injectable) - management of diabetes in the days of undercurrent illnesses - proper use and handling of needles and syringes.
Comissão Nacional de Incorporação de Tecnologias do SUS (CONITEC), 2018 [54]	Multidisciplinary health team having diabetes education experience. Type 1 diabetes education should be carried out by a specialist physician (endocrinologist) and a multidisciplinary health team.	Type 1 diabetes and parents. The educational program needs to be compatible with the level of cognitive development and adapted to the intellectual capacity of the child, adolescent, and family members.	Not provided.	Not provided.	Not provided.	Not provided.	Not provided.	Focus on self-management and should include the following educational topics: - healthy eating - carbohydrate counting - exercise - identification and treatment of hypoglycemia - insulin administration - intensive insulin therapy - tracking complications

### Environmental scan of other patient education programs for diabetes patients

Twenty cardiac rehabilitation centers were identified, and program managers were contacted by email to answer questions related to the educational component of the program. Of these, 2 (10%) responded. Both responders identified that their centers have education initiatives for people with diabetes. In summary, education is delivered every 2 weeks, with a duration of one-hour by a multidisciplinary team of healthcare providers through group lectures and interactive activities. Cognition and knowledge assessments are included. According to responders, the content of the education was not based on guidelines and topics covered included the following: diet, sedentary behavior, motivation, medication, disease limitations, and self-care. Although education initiatives are in place, no structured education program was reported.

### Assessment of information needs of patients identified by diabetes experts

Twenty Brazilian diabetes experts were contacted, of which 15 (75%) completed the instrument to assess the information needs of diabetes patients. This sample was comprised of 11 physicians, two nurses, one dietitian, and one psychologist. Results from this assessment are reported in Table 2. All 80 items were scored high, indicating that healthcare providers understand that diabetes patients have high information needs of essential topics related to their health condition. The item with the highest score was “What are the signs and symptoms of diabetes?” and “How do diabetes medicines act in the body?” was the item with the lowest score. Reading food labels was the area with the highest information needs scores, and cholesterol, triglycerides, and the Mediterranean diet pattern had the lowest scores. In regards to Diabetes College pillars, treat diabetes had the highest information needs reported and eat healthily had the lowest one. This information was used to organize the schedule of education so items that scored high were added in the first sessions, allowing patients patients to have their needs met as soon as they enter the program. Finally, when questioned about how they perceived diabetes patients would like this information to be delivered to them, (1) eight respondents identified that their patients prefer discussions with doctors or healthcare team, (2) seven respondents identified lectures and internet resources as their patients’ preferences, (3) six respondents identified a printed book, and (4) two respondents identified movies or videos that patients can watch at home.

**Table 2:** Results from the assessment of the information needs of patients identified by diabetes experts (*n* = 15)

Diabetes College Pillars	Area	Topics	Mean ± SD of item	Mean ± SD of area	Mean ± SD of pillars
Treat Diabetes	Overview of diabetes	1. What does the pancreas do, and what is the role of insulin?	4.53 ± 0.64	4.75 ± 0.26	4.61 ± 0.25
		2. What is diabetes and what happens in the body?	4.93 ± 0.26		
		3. What are the signs and symptoms of diabetes?	5.00 ± 0.00		
		4. What tests are used to diagnose diabetes?	4.53 ± 0.52		
	Management of blood sugar	5. What a glucometer is, how to read it, and when to use it?	4.90 ± 0.26	4.75 ± 0.21	
		6. What does the A1c test reveal about the blood sugar levels?	4.60 ± 0.62		
	Management of diabetes	7. How to manage diabetes?	4.80 ± 0.41	4.64 ± 0.13	
		8. What are the target levels for glycated hemoglobin?	4.60 ± 0.63		
		9. What are the target levels for blood pressure?	4.73 ± 0.46		
		10. What are the target levels of cholesterol?	4.47 ± 0.64		
		11. How to manage other factors that affect diabetes (e.g., depression, fitness and activity level, stress, and smoking)?	4.60 ± 0.63		
	Hypoglycemia	12. What are hypoglycemia and its signs and symptoms?	4.93 ± 0.26	4.73 ± 0.17	
		13. What are the risk factors and treatment for hypoglycemia?	4.66 ± 0.62		
		14. 14. How to prevent hypoglycemia?	4.80 ± 0.41		
		15. What is the impact of hypoglycemia on driving?	4.80 ± 0.41		
		16. 16. What is diabetes medical identification jewelry, and why to wear?	4.47 ± 0.52		
	Hyperglycemia	17. 17. What are hyperglycemia and its signs and symptoms?	4.67 ± 0.49	4.62 ± 0.12	
		18. What is the impact of hyperglycemia?	4.66 ± 0.49		
		19. What are the risk factors and treatments for hyperglycemia?	4.73 ± 0.46		
		20. How to treat hyperglycemia when the patient is ill?	4.40 ± 0.74		
		21. How to prevent hyperglycemia?	4.67 ± 0.49		
		22. What is the impact of hyperglycemia on exercise?	4.60 ± 0.63		
	Health problems associated with diabetes	23. What is the impact (complications) of diabetes on your body?	4.73 ± 0.46	4.49 ± 0.36	
		24. How to prevent diabetes-related complications?	4.67 ± 0.49		
		25. What tests can be performed to identify complications related to diabetes?	4.07 ± 0.59		
	Diabetes medicines	26. What are diabetes medicines?	4.20 ± 0.56	4.21 ± 0.29	
		27. How do diabetes medicines act in the body?	3.93 ± 0.80		
		28. Who can help patients with the management of diabetes medicines?	4.50 ± 0.52		
Get Active	Getting active and starting an exercise program	29. Why sitting less and moving more helps prevent diabetes?	4.73 ± 0.46	4.63 ± 0.11	4.59 ± 0.21
		30. How to spend less time sitting?	4.73 ± 0.46		
		31. What is physical activity, and how it helps manage diabetes?	4.60 ± 0.63		
		32. How to start exercising?	4.70 ± 0.49		
		33. What happens with blood sugar levels when someone is exercising?	4.50 ± 0.64		
		34. Which type of exercise diabetes patients should start?	4.50 ± 0.64		
	Types of exercise	35. What are aerobic exercise and its benefits?	4.27 ± 0.88	4.42 ± 0.11	
		36. How should the diabetes patient do his/her aerobic exercise?	4.53 ± 0.64		
		37. What are resistance training and its benefits?	4.40 ± 0.83		
		38. How should the diabetes patient do his/her resistance training?	4.47 ± 0.64		
	Exercise safety	39. How to prevent hypoglycemia?	4.87 ± 0.35	4.67 ± 0.30	
		40. What to eat before exercise, and what should be avoided (smoking and alcohol)?	4.87 ± 0.35		
		41. How take care of feet for exercise?	4.80 ± 0.41		
		42. How to prevent muscle and joint injuries while exercising?	4.33 ± 0.72		
		43. How to exercise safely in hot and cold weather?	4.30 ± 0.70		
		44. How to exercise safely with certain medical problems?	4.87 ± 0.35		
Eat Healthy	Nutrition basics	45. What are the four food groups?	4.53 ± 0.64	4.40 ± 0.11	4.28 ± 0.21
		46. What foods have carbohydrates, proteins, and fats?	4.40 ± 0.47		
		47. How should diabetes patient plan their meals?	4.40 ± 0.47		
		48. What are the options for healthy snacks?	4.27 ± 0.51		
	Mindful eating and intuitive eating	49. What is mindful eating and intuitive eating and how they can help diabetes patients manage their condition?	4.27 ± 0.51	4.27 ± 0.00	
	Fiber and glycemic index	50. What are the types of fiber?	4.13 ± 0.65	4.17 ± 0.07	
		51. How much fiber a patient needs to manage his/her diabetes?	4.20 ± 0.52		
		52. How can they get more fiber in a day?	4.20 ± 0.65		
		53. How much fiber there is in plant foods?	4.07 ± 0.75		
		54. What is glycemic index?	4.13 ± 0.51		
		55. How can low glycemic index foods help the management of diabetes?	4.27 ± 0.51		
		56. What factors affect the glycemic index of foods?	4.20 ± 0.52		
	Cholesterol, triglycerides and the Mediterranean diet pattern	57. What are cholesterol and which types?	4.13 ± 0.51	4.13 ± 0.05	
		58. How does the Mediterranean diet help manage diabetes?	4.07 ± 0.70		
		59. How to eat a Mediterranean diet?	4.20 ± 0.56		
		60. What are triglycerides and how to control their levels?	4.13 ± 0.83		
	Blood pressure and the DASH diet pattern	61. What is the link between diabetes and high blood pressure?	4.07 ± 0.50	4.34 ± 0.25	
		62. What are the hidden sources of sodium?	4.60 ± 0.63		
		63. How can the DASH eating pattern lower blood pressure?	4.07 ± 0.96		
		64. What amount of sodium is ok for diabetes patients?	4.47 ± 0.83		
		65. What else can be done to lower blood pressure?	4.47 ± 0.83		
	Reading food labels	66. What are the different types of nutrition information on a food label?	4.87 ± 0.35	4.87 ± 0.00	
Feel Well	Managing feelings and diabetes burnout	67. How to manage feelings about having diabetes?	4.80 ± 0.41	4.67 ± 0.12	4.39 ± 0.23
		68. What are diabetes burnout and its signs and symptoms?	4.60 ± 0.51		
		69. How to prevent and deal with diabetes burnout?	4.60 ± 0.51		
	Sleep, stress, anxiety, and depression	70. What does it mean to have a ‘good night sleep’ and how to achieve it?	4.27 ± 0.51	4.27 ± 0.00	
		71. What is sleep apnea?	4.20 ± 0.65		
		72. What is stress, and how to manage it?	4.20 ± 0.65		
		73. What is anxiety, and how to manage it?	4.27 ± 0.51		
		74. What is depression, and how to manage it?	4.20 ± 0.65		
	A healthy relationship	75. What is a healthy relationship?	4.20 ± 0.52	4.40 ± 0.22	
		76. How can diabetes impact sexual intimacy?	4.60 ± 0.51		
Take Control	Vision, goals and action plans	77. What is self-management and to self-manage diabetes?	4.60 ± 0.27	4.53 ± 0.05	4.53 ± 0.05
		78. How to define a vision, set goals, and build action plans to change life?	4.53 ± 0.36		
		79. How to problem-solve to manage diabetes?	4.47 ± 0.43		
		80. How to review action plans?	4.53 ± 0.92		

SD indicates standard deviation

Likert type scale ranged from 1 = *really not important* to 5 = *very important*

### Patient focus group

Six focus groups were held with 32 patients in total. Male and female patients were equally represented, 70% with a diagnosis of type 2 diabetes and one participant with prediabetes, with 80% receiving less than 5 Brazilian minimum wages per month (which corresponds to US$1200) and 75% with lower educational attainment (i.e., less than high school). Some of these characteristics are consistent with previous population-based studies [52–54]. The education importance was highlighted in all themes and the need for information access related to each topic. Participant quotes are attributed to pseudonyms. Patients’ experiences living with diabetes or prediabetes were grouped into six themes, all related to the pillars of Diabetes College (named treat diabetes, get active, eat healthy, feel well, and take control) and described below.

#### Self-management

One theme that emerged was self-management (related to “Take Control” pillar), as patients were generally aware of the importance of self-managing their disease and noted that the diagnosis of diabetes comes with a lot of responsibility. However, patients felt a lack of support to deal with multiple aspects of the disease and were not always motivated to do it.

*“Life with diabetes is a life with discipline; if you want to live well and not have complications, you have to follow a routine of self-care. […] I feel it is a lonely disease because people who do not have the disease do not know how tired the routine can be and what is involved.” “*Julie”, between the ages of 30–40 years old, with a diagnosis of type 1 diabetes for 10 years.

#### Physical activity

Another theme that emerged from the focus groups was physical activity, which was related to the “Get Active” pillar.. Most patients were aware of the importance of physical activity to control their disease but identified difficulties in establishing an exercise routine or adhering to an exercise program.

*“I joined a gym, but I do not go often. I know it is important [to my health], but it is hard to attend. When I am exercising, I have questions about what to eat before, during, and after the exercise routine. I am afraid to not feel well because my glucose levels go down too fast. I would like to know if there is an ideal type of exercise for diabetes.” “*Mary”, between the ages of 20–30 years old, with a diagnosis of type 1 diabetes for 14 years.

#### Eating habits

Another theme was eating habits (“Eat Healthy” pillar), being named the most common issue with diabetes for several patients. Patients frequently mentioned they try to eat healthily, and they know the importance of this behavior to their health. However, they reported that they cann.

ot always follow a healthy eating routine for reasons including financial problems, lack of knowledge, and lack of support. Some patients mentioned they are curious about alternative ways of eating to improve their health conditions.

*“It is hard to cut some types of food and vegetables and fruits are not the same as they were in the past. I am not sure that the food I buy is healthy*.” “Lucas”, between the ages of 60–70 years old, with a diagnosis of type 2 diabetes for 14 years.

#### Diabetes medication

A theme that emerged from the focus groups was the difficulty patients had to access their medications (“Treat Diabetes” pillar). In addition, several patients reported they are not confident about their prescribed medication, and sometimes they do not follow the doctor’s recommendations. There are also questions regarding the use of insulin.

*“My wife is the one that controls my medications. I believe that little information about the use of medications was explained to me, and I have questions regarding use and storage.” “*Marc” between the ages of 40–50 years old, with a diagnosis of type 2 diabetes for 14 years.

#### Psychosocial wellbeing

Another important theme that emerged from focus groups was psychosocial wellbeing (“Feel well” pillar), specifically anxiety, stress, depression, and discouragement. Patients reported that the diagnosis of diabetes comes with these feelings, and this consequently affects disease control and the ability to self-manage. Family problems and lack of support were also mentioned as contributors to unpleasant feelings.

*“I believe psychosocial wellbeing influences the control of my disease. I was always anxious and already had depression, which influences my glucose levels. I feel my anxiety increased with the diagnosis of the disease.” “*Rose” between the ages of 50–60 years old, with a diagnosis of type 2 diabetes for 4 years.

#### Sleep

Another theme was sleep (“Feel well” pillar). Most patients mentioned they have sleep problems, and they were aware of the relationship between sleep and diabetes. They also mentioned a lack of information and support related to this issue.

*“I do not sleep well. Usually, [I sleep] 5 hours per night. I do not know why. Maybe [due to] anxiety, maybe family issues. I do not speak with my doctor about this because he will not understand*.” “Albert” between the ages of 60–70 years old, with a diagnosis of type 2 diabetes for 9 years.

### Translation and cultural adaptation of the patient guide

The first step was the preparation and involved the initial work carried out before the translation work began. Some of the critical components of this step comprised recruitment of key persons for the project (including a certified translator and consultants to review translated materials) and identifying the target audience based on results from the needs analysis. The second step was the translation, in which the original language (English) was translated to Brazilian Portuguese. There is a general agreement in the existing translation guidelines regarding the need for more than one translation carried out independently; however, due to budget restrictions, only one translation was developed. The third step was back-translation, which involved translating the new language version (Brazilian Portuguese) back into the original language (English). For practical purposes, a more literal (not conceptual) back-translation was performed by key persons (native speakers of the target language and fluent in the source language) identified in the first step. This step is considered a quality-control step [46], and it could demonstrate the quality of the translation since the same meaning was derived when the translation was moved back into the source language. Following back-translation, the back-translation review was performed. This fourth step is considered one of the most critical components of the cultural adaptation and, in summary, compares the back-translated version with the original to highlight and investigate discrepancies between versions. This step allowed us to identify changes in the content that were generated based on cultural aspects or errors.

The harmonization step aims to detect and deal with any translation discrepancies between different language versions, thus ensuring conceptual equivalence and cultural adaptation between the source and the target language version. It provides an additional quality-control step and further ensures that data can be safely aggregated. First, the translated guide was reviewed by the key in-country consultants. Second, chapters were given to a team of healthcare providers and experts in specific areas (for example, a Brazilian dietitian received the nutrition chapters) to check for applicabily of information in the context of their patients. They were asked to provide justification if changes were suggested. To conclude this fifth step, key in-country consultants reviewed all chapters once more. Twelve of the twenty chapters of this guide were culturally adapted, and the main changes are described in Table 3.

**Table 3:** Cultural adaptations performed on the Brazilian Portuguese version of the Diabetes College Patient Guide

Section	Culturally Adapted?	Changes
Treat Diabetes	Yes Chapters 1–7	• Terms and nouns adapted to the Brazilian culture. • Units changed to reflect the Brazilian system. • Change the name of medications to reflect the names in Brazil (some medications were excluded because they are not commercialized in Brazil). • Management of glucose and tests were updated to current practice in Brazil. • Some management programs for Diabetes were excluded because they are not available in Brazil.
Get Active	Yes Chapters 1–3	• Terms and nouns adapted to the Brazilian culture. • Types of sports were changed to reflect Brazilian culture (e.g., golf was replaced by soccer). • Borg Scale Original was changed to the modified version that is currently used in Brazil. • Glycemic values for exercise: changed to the ones recommended by the Brazilian Society of Diabetes. • Air quality: pictures with references from Brazil were included. • Emergency contact: changed to the Brazilian health emergency service number (SAMU 192).
Eat Healthily	Yes Chapters 1 and 4	• Terms and nouns adapted to the Brazilian culture. • Canada’s Food Guide was substituted by the Brazilian one. • Foods that are not available in Brazil were excluded. • Consumption of fiber was changed to reflect what is recommended by Brazilian dietitians. • LDL and HDL target levels were updated to reflect Brazilian guidelines. • Links of nutritional programs only available in English were excluded.
Feel Well	Yes Chapters 1 and 2	• Terms and nouns adapted to the Brazilian culture.
Take Control	Yes Chapter 1	• Terms and nouns adapted to the Brazilian culture.
Toolbox	Yes	• Borg Scale was changed to reflect the one currently used in Brazil. • Website to track walk distances was updated. • English resources were updated to Brazilian ones.

*LDL* low density lipoproteins, *HDL* high density lipoproteins

The sixth step was proofreading in which all consultants involved in the project and patients read the final version of the guide and identified problems and typos. All materials were written in plain language aiming a reading level of 5 to eliminate possible barriers related to limited education and income After this last step, the guide was finalized and ready to be used by patients.

### Design of the patient education program

The patient guide is the primary tool of the patient education program; however, it is not the only material that will be used to deliver education to diabetes patients. Based on the needs analysis, the design of the program is described in Table 4, following the reporting guidelines for behavior change interventions developed by WIDER [37]. Theory was incorporated in the design (guide and future lectures) by encourage learners to reflect on their own experiences, create learning strategies and action plans, and actively participate in the learning process. In addition, design was developed working “backwards” from outcomes to the other elements (e.g. content, teaching and learning experiences, assessment and evaluation). As self-management is the main outcome we want patients to achieve, all other elements were developed based on this goal. The schedule for each educational session is described in Table 5.

**Table 4:** Design of the patient education program following the reporting guidelines for behavior change interventions developed by WIDER

Characteristic	Description
Characteristics of those delivering the education	A multidisciplinary team of healthcare providers will deliver the education program, including physiotherapists, physicians, pharmacists, dietitians, and physical educators. These professionals attended capacity building sessions to understand patient and family education and counseling concepts and to increase knowledge, skills, and resources regarding adult learning principles and diabetes care.
Characteristics of the recipients	This education program was designed for adults with diabetes or prediabetes.
The setting	This program will be delivered as part of an Exercise and Lifestyle Education Program in two cities in Brazil. Classes will take place in a private room with a maximum of 10 patients in each class, preferably prior to the exercise component of the program.
Mode of delivery	Sessions will include all four categories of teaching methods reported in the literature (instructor-centered, interactive, individualized techniques, and experiential learning). All participants will receive the patient guide and will be oriented to read different chapters each week.
Frequency	It will be offered through 18 education classes, and the schedule of these classes is described in Table 5. These classes will be delivered as part of a 12-week education intervention: in the first 4 weeks with two classes per week and the remaining with 1 class per week.
Duration	Educational sessions will be 30 min long, except for the orientation (1st session) and the diabetes medication session, which will be 1-h long. This change was based on the results of the needs analysis.
Adherence to delivery intervention	A healthcare team member will register the attendance of participants, as well as their family members, at the starting of each class. Also, a lesson plan was developed for each class, which states program learning outcomes, session learning outcomes, the structure of classes, and suggested activities.
Detailed description of the intervention content	The content of each class is based on the five pillars of Diabetes College. The classes were structured based on the needs analysis. Topics are described in Table 5.

**Table 5:** Schedule of educational sessions for the Diabetes College program in Brazil

Class	Topic	Guide Sections and Chapters	Pages
1	Welcome to the program	Section: Get Active Chapter 1: Getting Active & Starting an Exercise Program	107–114
		Resources	266–267,269,284
2	Your Exercise Safety	Section: Get Active Chapter 2: Types of Exercise	115–134
		Chapter Resources	268,273–276
3	Your Exercise Safety	Section: Get Active Chapter 3: Your Exercise Safety	135–154
		Resources	270–272,277–278, 286–289
4	Manage Your Blood Sugar	Section: Treat Diabetes Chapter 2: Manage Your Blood Sugar	15–22
		Resources	286
5	Hypoglycemia/Hyperglycemia	Section: Treat Diabetes Chapters 4: Low Blood Sugar (Hypoglycemia) Chapter 5: High Blood Sugar (Hyperglycemia)	35–50
6	Resistance Exercise – Day 1	Section: Get Active Chapter 2: Types of Exercise	124–133
		Resources	285
7	Resistance Exercise – Day 2	Section: Get Active Chapter 2: Types of Exercise	124–133
8	Know and Control Cardiovascular Disease Risk Factors	Section: Treat Diabetes Chapter 3: Manage Your Diabetes	23–34
9	Health Problems Caused by Diabetes	Section: Treat Diabetes Chapter 6: Health Problems with Diabetes	51–72
10	Vision, Goals and Action Plans	Section: Take Control Chapter 1: Vision, Goals and Action Plans	247–264
		Resources	294–296
11	Emotional Well Being – Day 1	Section: Feel Well Chapter 1: Managing Your Feelings and Diabetes Burnout	225–230
12	Understanding Diabetes Medicines	Section: Treat Diabetes Chapter 7: Diabetes Medicines	73–106
		Resources	265
13	How Food Affects Blood Glucose	Section: Eat Healthily Chapter 1: Nutrition Basics	155–166
		Resources	290–293
14	Mindful Eating and Intuitive Eating	Section: Eat Healthily Chapter 2: Mindful Eating and Intuitive Eating	167–172
15	How Food Affects Blood Pressure and Cholesterol	Section: Eat Healthily Chapter 3: Fiber and Glycemic Index Chapter 4: Cholesterol, Triglycerides, and the Mediterranean Diet Pattern Chapter 5: Lower Your Blood Pressure with the DASH Diet Pattern	173–212
16	Emotional Well Being – Day 2	Section: Feel Well Chapter 2: Sleep, Stress, Anxiety, and Depression Chapter 3: A Healthy Relationship	231–246
17	Learning to Read the Food Label	Section: Eat Healthily Chapter 6: Learn How to Read Food Labels	213–224
18	Graduation	Resources	297–298

### Assessment of the patient education program

A randomized controlled trial (ClinicalTrial.org identifier: NCT03914924) is currently ongoing to investigate the effects of an Exercise and Lifestyle Education Program on functional capacity and other outcomes in diabetes and prediabetes patients compared to an Exercise Program. Disease-related knowledge, health behaviors, and cardiometabolic health parameters are the secondary outcomes, and the tertiary outcomes are quality of life, program adherence, satisfaction about learning tools and structured of the program, and six-month related diabetes morbidity. All these outcomes were carefully chosen and the program was designed to achieve these endpoints. For instance, the tool that will assess disease-related knowledge (named Diabetes Education Questionnaire; DATE-Q) [55] has been developed and psychometrically validated in Brazilian-Portuguese based on the education delivered to patients [56]. It is hypothesized that the Exercise and Lifestyle Education Program, which is delivering the educational program described in this article, besides exercise, will promote significantly better outcomes than the Exercise Program (with no educational intervention).

## Discussion

This article described all development phases of an education program for diabetes and prediabetes patients as well as their families in Brazil. This new program consists of 18 educational sessions strategically mapped and sequenced to support the program learning outcomes and a patient guide with 17 chapters organized into five sections and matching weekly lectures. Positive aspects of this program include the following: based on needs analysis results from multiple groups (patients, experts, and other programs); translated following rigorous steps and culturally adapted to the Brazilian population; topics structured from simplest to the most complex; instructional strategy and design comprising learning activities, learning assessments and learning resources and materials; all four categories of teaching methods included (instructor-centered, interactive, individualized techniques and experiential learning); and, capacity building sessions to increase knowledge, skills, and resources of instructors.

Overall, results from this study showed that not all available guidelines for the care of individuals with diabetes in South America recommend education, even with the abundance of evidence supporting its benefits and an urge to promote this practice [57]. When listed, educational strategies are not fully characterized, which can preclude healthcare providers from incorporating this in the care of their patients. The lack of structure in these programs is also true when assessing the information provided by the two respondents from the environmental scan and other studies published in Brazil, [58, 59] where patient education is not delivered or, if available, not structured.

It is known that individuals with diabetes have a higher need for information compared with people with other chronic diseases [60, 61]. This has been confirmed by diabetes experts that rated their patients’ information needs high in all areas. Studies have also shown that this population has high needs to learn about medications, [60–62] which was also identified by experts and patients during focus groups in this study. In addition, psychosocial wellbeing was a theme extracted from the focus groups and has been often described in studies assessing information needs of individuals with diabetes as an important educational topic [61].

This needs analysis was an essential aspect of this study because it allowed us to develop a vision for a patient and family education program from information gathered from four activities. These activities were very comprehensive and involved multiple assessments with different populations, including patients, other programs, and diabetes experts. Synthesizing the information gathered from the needs analysis allowed us to develop a vision for a patient and family education program that includes: a variety of topics and modes of delivery that are relevant to diabetes patients; information to help patients to make informed decisions about their health; and information that is also consistent across the team members and is supported by evidence.

Another key strength of the program developed is the empirically derived and conceptually congruent intervention. The educational intervention proposed was developed on the principles of the following theories: constructivist learning theory, adult learning theory, the health action process approach (HAPA) model, and prescriptive model. Patient guide and lectures were designed to include important components of these theories (e.g. weekly action plans, encourage patients to share their experiences, learmning building up from previous experiences). In addition, because diabetes is considered a chronic condition, the intervention was designed to promote self-management and incorporated self-management skills. Reviews of the effectiveness of chronic disease management interventions indicate that interventions based on behavioral change models are more likely to be effective than those that are not [63].

Although this educational intervention was theory-driven and evidence-based, other important components should be evaluated such as process (how the program is delivered best to this population), and acceptability of the program to those who might participate. This study focus on content and these other characteristics should form the basis of future studies. In addition, the effectiveness of the proposed model must be investigated. As described elsewhere, a randomized controlled trial is in progress to investigate the effects of an Exercise and Lifestyle Education Program with exercise and educational interventions on functional capacity as primary outcomes in adults with diabetes and prediabetes. Our efforts are concentrated on this investigation whose participants’ recruitment is ongoing and is expected to be concluded in 2 years.

Caution is warranted when interpreting these results. First, the program was designed for individuals with diabetes and prediabetes; however, only one individual with prediabetes has participated in this study. Second, the environmental scan was only completed by 2 programs, so generalizability is limited. Moreover, this was a small sample size for diabetes experts, which limits generalizability. Third, information needs of patients was identified by diabetes experts. Report of information needs by patients themselves and congruences and differences identified by these groups should be highlighted in a future study. In addition, the sample of experts is heterogeneous in terms of disciplines but not proportionate. Forth, in the analysis of themes that emerged from focus groups, patients were not involved in picking or validating themes; however, each theme was reviewed by the research team to ensure that it reflected both its associate coded extracts and the entire data set. Finally, no structured method of analysis for the harmonization step was used; however, healthcare providers and experts were contacted to provide more information about their changes and overall comments if needed.

## Conclusion

In conclusion, the development of the patient education program described in this article followed several steps in order to be applicable to the Brazilian population living with diabetes or prediabetes and fit to these patients’ needs and local guidelines.

This program may be a powerful tool to promote behavior change and self-management for people living with diabetes or prediabetes in Brazil, which effectiveness will be tested by further research. Besides, programs focus on diabetes care can use this model to educate their patients or follow these steps to develop their own education programs. Meeting individual needs may require a sustained effort, diverse strategies based on resources available, and additional research to explore the impact of these strategies on outcomes, feasibility of program design, and patients’ satisfaction.

## Supplementary Information

**Additional file 1.** Environmental Scan Questions based on WIDER (generated by authors).

**Additional file 2.** Tool used for the assessment of information needs of patients identified by diabetes experts (generated by authors).

**Additional file 3.** Patient Focus Group Guide.

## Data Availability

The datasets used and analyzed during the current study are available from the corresponding author on reasonable request.

## Abbreviations

CR: Cardiac rehabilitation
DAB: Diabetic Association of Bangladesh
GHb: gamma-hydroxybutyrate
HAPA: Health Action Process Approach
HbA1c: glycated hemoglobin
NICE: National Institute for Health and Care Excellence
WIDER: Workgroup for Intervention Development and Evaluation Research
