# Supplementary material for: Patient education program for Brazilians living with diabetes and prediabetes: findings from a development study
Source: BMC Public Health. 2021 Jun 26;21:1236. doi: 10.1186/s12889-021-11300-y (PMC8236150; doi:10.1186/s12889-021-11300-y)
Supplement: Supplementary file 1 — Additional file 1. Environmental Scan Questions based on WIDER (generated by authors). [file 12889_2021_11300_MOESM1_ESM.docx]

Additional File 1: Environmental Scan Questions based on WIDER (generated by authors)

Do you have a structured education intervention as part of your program for people living with diabetes?

If yes, please answer the following 8 questions:

1. Who are the providers delivering the education intervention? Are they trained in adult learning principles?
2. Who are the patients receiving the education intervention? Any inclusion or exclusion criteria?
3. Where is the education intervention delivered (i.e. setting)?
4. How is the education intervention delivered (i.e. mode of delivery)?
5. What is the intensity of the education intervention delivered (i.e. how many sessions)?
6. What is the duration of the education intervention delivered (i.e. how long is your program overall and per session)?
7. Do you keep track of patients’ adherence to the program? If yes, how?
8. Please describe the content delivered in detail.
