# Supplementary material for: Patient education program for Brazilians living with diabetes and prediabetes: findings from a development study
Source: BMC Public Health. 2021 Jun 26;21:1236. doi: 10.1186/s12889-021-11300-y (PMC8236150; doi:10.1186/s12889-021-11300-y)
Supplement: Supplementary file 2 — Additional file 2. Tool used for the assessment of information needs of patients identified by diabetes experts (generated by authors). [file 12889_2021_11300_MOESM2_ESM.docx]

Additional file 2: Tool used for the assessment of information needs of patients identified by diabetes experts

Rate the importance of each topic to increase your patients’ knowledge about diabetes

|  | **Really not important** | **Not important** | **Neutral** | **Important** | **Very Important** |
| --- | --- | --- | --- | --- | --- |
| 1. What does the pancreas do, and what is the role of insulin? |  |  |  |  |  |
| 2. What is diabetes and what happens in the body? |  |  |  |  |  |
| 3. What are the signs and symptoms of diabetes? |  |  |  |  |  |
| 4. What tests are used to diagnose diabetes? |  |  |  |  |  |
| 5. What a glucometer is, how to read it, and when to use it? |  |  |  |  |  |
| 6. What does the A1c test reveal about the blood sugar levels? |  |  |  |  |  |
| 7. How to manage diabetes? |  |  |  |  |  |
| 8. What are the target levels for glycated hemoglobin? |  |  |  |  |  |
| 9. What are the target levels for blood pressure? |  |  |  |  |  |
| 10. What are the target levels of cholesterol? |  |  |  |  |  |
| 11. How to manage other factors that affect diabetes (e.g., depression, fitness and activity level, stress, and smoking)? |  |  |  |  |  |
| 12. What are hypoglycemia and its signs and symptoms? |  |  |  |  |  |
| 13. What are the risk factors and treatment for hypoglycemia? |  |  |  |  |  |
| 14. 14. How to prevent hypoglycemia? |  |  |  |  |  |
| 15. What is the impact of hypoglycemia on driving? |  |  |  |  |  |
| 16. 16. What is diabetes medical identification jewelry, and why to wear? |  |  |  |  |  |
| 17. 17. What are hyperglycemia and its signs and symptoms? |  |  |  |  |  |
| 18. What is the impact of hyperglycemia? |  |  |  |  |  |
| 19. What are the risk factors and treatments for hyperglycemia? |  |  |  |  |  |
| 20. How to treat hyperglycemia when the patient is ill? |  |  |  |  |  |
| 21. How to prevent hyperglycemia? |  |  |  |  |  |
| 22. What is the impact of hyperglycemia on exercise? |  |  |  |  |  |
| 23. What is the impact (complications) of diabetes on your body? |  |  |  |  |  |
| 24. How to prevent diabetes-related complications? |  |  |  |  |  |
| 25. What tests can be performed to identify complications related to diabetes? |  |  |  |  |  |
| 26. What are diabetes medicines? |  |  |  |  |  |
| 27. How do diabetes medicines act in the body? |  |  |  |  |  |
| 28. Who can help patients with the management of diabetes medicines? |  |  |  |  |  |
| 29. Why sitting less and moving more helps prevent diabetes? |  |  |  |  |  |
| 30. How to spend less time sitting? |  |  |  |  |  |
| 31. What is physical activity, and how it helps manage diabetes? |  |  |  |  |  |
| 32. How to start exercising? |  |  |  |  |  |
| 33. What happens with blood sugar levels when someone is exercising? |  |  |  |  |  |
| 34. Which type of exercise diabetes patients should start? |  |  |  |  |  |
| 35. What are aerobic exercise and its benefits? |  |  |  |  |  |
| 36. How should the diabetes patient do his/her aerobic exercise? |  |  |  |  |  |
| 37. What are resistance training and its benefits? |  |  |  |  |  |
| 38. How should the diabetes patient do his/her resistance training? |  |  |  |  |  |
| 39. How to prevent hypoglycemia? |  |  |  |  |  |
| 40. What to eat before exercise, and what should be avoided (smoking and alcohol)? |  |  |  |  |  |
| 41. How take care of feet for exercise? |  |  |  |  |  |
| 42. How to prevent muscle and joint injuries while exercising? |  |  |  |  |  |
| 43. How to exercise safely in hot and cold weather? |  |  |  |  |  |
| 44. How to exercise safely with certain medical problems? |  |  |  |  |  |
| 45. What are the four food groups? |  |  |  |  |  |
| 46. What foods have carbohydrates, proteins, and fats? |  |  |  |  |  |
| 47. How should diabetes patient plan their meals? |  |  |  |  |  |
| 48. What are the options for healthy snacks? |  |  |  |  |  |
| 49. What is mindful eating and intuitive eating and how they can help diabetes patients manage their condition? |  |  |  |  |  |
| 50. What are the types of fiber? |  |  |  |  |  |
| 51. How much fiber a patient needs to manage his/her diabetes? |  |  |  |  |  |
| 52. How can they get more fiber in a day? |  |  |  |  |  |
| 53. How much fiber there is in plant foods? |  |  |  |  |  |
| 54. What is glycemic index? |  |  |  |  |  |
| 55. How can low glycemic index foods help the management of diabetes? |  |  |  |  |  |
| 56. What factors affect the glycemic index of foods? |  |  |  |  |  |
| 57. What are cholesterol and which types? |  |  |  |  |  |
| 58. How does the Mediterranean diet help manage diabetes? |  |  |  |  |  |
| 59. How to eat a Mediterranean diet? |  |  |  |  |  |
| 60. What are triglycerides and how to control their levels? |  |  |  |  |  |
| 61. What is the link between diabetes and high blood pressure? |  |  |  |  |  |
| 62. What are the hidden sources of sodium? |  |  |  |  |  |
| 63. How can the DASH eating pattern lower blood pressure? |  |  |  |  |  |
| 64. What amount of sodium is ok for diabetes patients? |  |  |  |  |  |
| 65. What else can be done to lower blood pressure? |  |  |  |  |  |
| 66. What are the different types of nutrition information on a food label? |  |  |  |  |  |
| 67. How to manage feelings about having diabetes? |  |  |  |  |  |
| 68. What are diabetes burnout and its signs and symptoms? |  |  |  |  |  |
| 69. How to prevent and deal with diabetes burnout? |  |  |  |  |  |
| 70. What does it mean to have a 'good night sleep' and how to achieve it? |  |  |  |  |  |
| 71. What is sleep apnea? |  |  |  |  |  |
| 72. What is stress, and how to manage it? |  |  |  |  |  |
| 73. What is anxiety, and how to manage it? |  |  |  |  |  |
| 74. What is depression, and how to manage it? |  |  |  |  |  |
| 75. What is a healthy relationship? |  |  |  |  |  |
| 76. How can diabetes impact sexual intimacy? |  |  |  |  |  |
| 77. What is self-management and to self-manage diabetes? |  |  |  |  |  |
| 78. How to define a vision, set goals, and build action plans to change life? |  |  |  |  |  |
| 79. How to problem-solve to manage diabetes? |  |  |  |  |  |
| 80. How to review action plans? |  |  |  |  |  |
