# Supplementary material for: Patient education program for Brazilians living with diabetes and prediabetes: findings from a development study
Source: BMC Public Health. 2021 Jun 26;21:1236. doi: 10.1186/s12889-021-11300-y (PMC8236150; doi:10.1186/s12889-021-11300-y)
Supplement: Supplementary file 3 — Additional file 3. Patient Focus Group Guide. [file 12889_2021_11300_MOESM3_ESM.docx]

Additional file 3: Patient Focus Group Guide

Focus group objective: To identify the main educational needs of patients with diabetes and prediabetes and, from this, develop an education program for Brazilians living with these conditions.

**INITIAL CONSIDERATION**

1. Number of participants per group: 10 patients with diabetes and/or prediabetes will be invited to the focus group to achieve at least 6 to 8 participants per group, and the goal of the focus group should be explained during the phone call invitation as well as encouraging the attendance.
2. Number of groups to be conducted: 3 groups will be held in each of the two Brazilian cities.
3. Participants' characteristics: the groups should be heterogeneous in terms of (a) time of disease diagnosis, (b) sex, (c) obesity (obese and non-obese), and (d) insulin-treated and insulin non-treated.
4. Duration: 90 minutes.
5. Procedures:

- The main moderator will introduce himself or herself to the group and reinforce the goal of the focus group.

- The patients will be asked to sign the consent form and the authorization form agreeing with the use of image and voice sound for research purposes.

- Those who give up participating in the focus group will not sign the consent form and should leave the room at any time before the discussion starts.

- The research team will hand over the badge to participants and put theirs on it.

- As an introduction part, the participants will be greeted with a snack so that they can get to know each other and feel more comfortable with each other and the research team. This introduction step will take for up to 10 minutes.

- During this introduction part, the participants will be asked to self complete a form to clinical and sociodemographic data collection, and the moderators should make a brief analysis of the responses on it to know the group profile.

- After that, the main moderator will give an initial speech and presentation (detailed below).

- The main moderator will start the discussion by following the guiding questions (described below).

- The main moderator will conclude the group activities.

**FOCUS GROUP LEADERS**

The focus groups will be conducted by at least three research team members, including:

- Two moderators: one main and one assistant

- One recorder

The **moderators** have three main responsibilities in conducting the focus group:

1. To ensure that participants sufficiently address the main topic
2. To encourage the non-talkative participants, those who are speaking less compare to their counterparts, to contribute to the discussion
3. To support the group discussion and facilitate the interaction between the participants

Summary, the moderators manage the discussion direction while moderate the group dynamics during the session.

The **recorder** also has three main responsibilities during the focus group session:

1. To manage the audio recorders to ensure its property quality
2. To write notes during the session to ensure that moderators can focus on group discussions and dynamics, including the following procedures: (a) to write the name and time that each participant starts their speech in the recording to facilitate the data analysis, (b) to facilitate the audio marking it is suggested that the assistant moderator pronounce the first name of the participant who is speaking previously his or her speech if the participant forgets it.
3. To share a summary of the discussion at the end of the session to participants' approval.

**INITIAL SPEECH AND PRESENTATION:**

1. Welcome.
2. Moderators and recorder's introduction.
3. Focus group's objectives explanation.
4. Focus group's rules explanation as well as each member of the research group function.
5. Encourage all the participants to express their points of view and experiences during the discussion.
6. Clarification that all shared experiences will be only used for the development of an education program for adults with diabetes and prediabetes.
7. Confidentiality of all information provided's explanation.

Speech suggestion:

*"Hello, welcome to this focus group. Firstly, we would like to thank you all for coming here today and for agreeing to share your experiences about how to live with diabetes or prediabetes looks like, including the difficulties, limitations, and expectations in managing these conditions. My name is ............, and I am going to moderate our discussion, along with the ......... (assistant moderator). We will also have the support of the ............. (recorder) who will be responsible for recording our discussion and taking note of the leading sharing topics. Each one of you has expressed interest in the subject to be discussed today, taking the time to come here to share your thoughts and opinions with us. We are here to listen to you all. The information shared will be used to help us in developing an educational program for adults with diabetes and prediabetes living in Brazil.*

*Your participation is entirely voluntary, and your identity and all information provided by you will be kept confidential by the research team. Although we will use your first name during this discussion, the focus group final reports will not contain any names to ensure participants' confidentiality."*

**BASIC RULES FOR DISCUSSION –** **AGREEMENT**

1. Everyone's privacy will be preserved, so the shared experiences and opinions must stay between the participants.
2. There are no right or wrong answers. Everyone should share their experiences and opinions even if they are different from the others because all of them are important.
3. Please say your first name previously to each time you are going to speak.
4. Only one participant should speak at a time with any order, and everyone will listen to all discussion topics. In this way, everyone will be able to listen carefully to each other's speech.
5. The moderators' role is to ask questions following the discussion guide and listen to the speeches. The discussion should be between the participants.

Speech suggestion:

*"During the discussion, there are some basic rules that we need to keep in mind to help the maintenance of comfort and privacy of all group participants. We would like to ask you to stay here everything that is being said in this focus group with respect to everyone's privacy on personal experiences and points of view that will be discussed by the group.*

*There are no right or wrong answers to any of the questions asked. There are only different experiences and points of view. Please feel free to share your thoughts, even if they are different from what others have said. It is important to listen to the full range of ideas and experiences that you all have.*

*Please try to avoid talking at the same time as someone else. We do not want to miss any of your comments, and if multiple participants are talking at the same time, the recording will be distorted.*

*Remember also that we are interested in both positive and negative comments and experiences, both are useful and important for us. As moderators, our main role is to ask questions and then listen and guide the discussion. We will not be participating in the discussion. You all can make yourselves comfortable and free to ask other questions related to the discussion topic.*

*At this point, I would like to check that everyone has signed the consent form. If you have not yet signed and would like to participate in the focus group, please sign and give it to us before we start the discussion. If someone is not interested in participating anymore, I would like to ask you, and please leave the room as well as I would like to thank you for coming".*

**DISCUSSION GUIDING QUESTIONS**

1. ***How is your life with diabetes or prediabetes?***

We believe that this question will guide the discussion through the difficulties and challenges of participants living with these conditions. We will consider the statements emerged related to the topics to find out how exactly this difficulty impact in the participant's life and the key points that should be explored are as follow: (a) food/diet, (b) physical exercise, (c) use of medications, (d) sleep and, (e) stress/anxiety and depression. If these aspects of life with diabetes or prediabetes will be not mentioned, try to direct them using open questions, e.g., *"What about food? What would you have to say?"* or *"What do you think about exercising?"*

**Tips!**

- It could be helpful to use metaphors to encourage participants to express their experiences.

- Use questions like *"For what...?"* and *"What do you mean..."*

- If it is noticed that someone is not participating, you may ask: *"Does anyone else feel in the same way?"* or *"Does anyone feel differently?"*. Besides, you can ask if anyone else has any experience in the aspect discussed to give the opportunity for others to speak. In this way, the other participants will have a cue to also participate in the conversation, and they will feel the urge to agree or disagree with each other.

- If it takes too long for someone to conclude his or her speech, think of the best way to guide his or her speech conclusion.

- If the discussion is going out of the key points, you can say something like this *"Thank you so much for your speech, remind me, so that the next opportunity to talk about it, you can continue ..."*

1. ***What do you think could be changed to improve your life with diabetes or prediabetes?***

**CONCLUSION**

1. The recorder will read the summary of the main points addressed.

2. The moderators will thank everyone for their participation and availability.

Speech suggestion:

*"As we conclude our time together, we want to make sure that we write down all the points that are important to you. For this, the recorder will read a summary of the main points addressed in the discussion. Please let us know if there is something else that we missed or some point which you would like to add.*

*Thank you for taking the time to come here today and share your point of view and experience with us. We will carefully analyze all this discussion to develop an education program for Brazilians living with considering your opinions. If you have any questions, feel free to ask us now or at any other time as you wish."*
